# Tooth Auto-Transplantation in Patients with Cleft Lip and/or Palate: A Systematic Review and Preliminary Clinical Protocol Proposal

**DOI:** 10.3390/jcm15155829

**Published:** 2026-07-25

**Authors:** Mohamad Awos Sulaiman, Austėja Rudytė, Eglė Ulbinaitė, Bohdan Haltsev, Yaman Sulaiman, Gintaras Juodžbalys, Arūnas Vasiliauskas

**Affiliations:** 1 Faculty of Odontology, Lithuanian University of Health Sciences, A. Mickevičiaus Str. 9, LT-50161 Kaunas, Lithuania; austeja.rudyte@stud.lsmu.lt (A.R.); egle.ulbinaite@stud.lsmu.lt (E.U.); bohdan.haltsev@stud.lsmu.lt (B.H.); 2 Clinic of Dental and Oral Pathology, Faculty of Odontology, Medical Academy, Lithuanian University of Health Sciences, Eivenių Str. 2, LT-50161 Kaunas, Lithuania; yaman.sulaiman@stud.lsmu.lt; 3 Department of Oral and Maxillofacial Surgery, Faculty of Odontology, Medical Academy, Lithuanian University of Health Sciences, LT-50161 Kaunas, Lithuania; gintaras.juodzbalys@lsmu.lt; 4 Department of Orthodontics, Faculty of Odontology, Medical Academy, Lithuanian University of Health Sciences, LT-50166 Kaunas, Lithuania; arunas.vasiliauskas@lsmu.lt

**Keywords:** tooth auto-transplantation, cleft lip and/or palate, secondary alveolar bone graft, surgery, orthodontics, endodontics, periodontics, systematic review

## Abstract

**Background**: Tooth auto-transplantation (TAT) is a surgical procedure in which a person’s own tooth is extracted and repositioned in the recipient site. The aim of the study is to evaluate the clinical outcomes, treatment strategies, and methodological characteristics, and to propose a preliminary management algorithm of TAT in patients with cleft lip and/or palate (CLP). **Methods**: A search of the literature was conducted in PubMed, Google Scholar, ClinicalKey, Web of Science, and Cochrane Library databases until 21 June 2026. The systematic review was written according to Preferred Reporting Items for Systematic Reviews and Meta-analyses (PRISMA) guidelines. Risk-of -bias was assessed by the Joanna Briggs Institute (JBI) tool. **Results**: Five case series and three case reports were included, with four “low” and four “moderate” JBI reporting-quality ratings (reflecting reporting completeness, not low risk of bias). They presented data about 23 patients and 27 TAT cases. The majority of patients (*n* = 16) received secondary alveolar bone graft with iliac bone. The most common donor teeth were mandibular premolars (*n* = 20). The most frequent recipient sites were the maxillary 2nd premolar (*n* = 9) and maxillary incisors (*n* = 15). Twenty-six teeth (96.3%) survived, and one tooth (3.7%) was extracted. **Conclusions**: TAT appears to be a promising treatment option in carefully selected CLP patients; however, further high-quality studies are needed with the application of our proposed algorithm.

## 1. Introduction

Cleft lip is defined as a facial deformity with malfusion of the upper lip occurring either uni- or bilaterally as morphologically complete or incomplete. In most cases, cleft palate is present in combination with cleft lip, which is a failure of the hard palate fusion during embryological development [1]. The most common craniofacial congenital anomaly in newborns is cleft lip and/or palate (CLP), with an estimated prevalence of 0.1% in the general population [2]. Despite the worldwide ethnic group variations, the approximate number of people born with orofacial clefts is about 1 in 600 to 800 live births [3]. Cleft lip with or without palate affects males approximately twice as often as females [4]. The aetiology of CLP is categorised into genetic and non-genetic (e.g., smoking and alcohol) causes during embryological facial development between weeks 4 and 8 [5,6]. The anomaly is associated with a variety of functional and aesthetic consequences, most commonly aplasia of the maxillary lateral incisors. Other dental anomalies such as supernumerary teeth, decayed teeth, microdontia, etc., often occur alongside [6].

In the 1950s, tooth auto-transplantation (TAT) was introduced for the management of tooth hypodontia in cases of CLP. This is a surgical procedure in which a person’s own tooth (usually premolars or third molars) is extracted from its original location while sustaining the periodontal ligament and is repositioned in the recipient site, which is usually the area of a missing or damaged tooth [7,8,9,10]. The procedure should be completed within 15 min to maintain the viability of the periodontal cells and followed by suturing or splinting of the autotransplanted tooth [9,11].

However, patients with CLP require a pre-operative bone augmentation procedure, which often involves an autologous bone graft. The most reliable and consistent method of augmentation of the bony alveolar cleft area is to harvest and utilise iliac bone, as it is a large source of osteogenic cancellous bone [12]. After TAT, orthodontic and endodontic interventions are often required as part of post-operative treatment, such as orthodontic appliances to stabilise the auto-transplanted teeth and root canal treatment (RCT) to stimulate further root development or prevent endodontic complications [13,14].

To the best of the authors’ knowledge, no systematic review has been published to date that focuses on the use of TAT in patients with CLP. The aim of this study is to gather available studies, critically evaluate patient characteristics, treatment strategies, and clinical outcomes, and propose a preliminary expert-informed clinical algorithm for patients with CLP undergoing TAT. Analysis of this treatment modality based on existing cases would significantly improve patient treatment outcomes and success.

## 2. Materials and Methods

### 2.1. The Protocol for the Systematic Review

The present systematic review was conducted according to the guidelines of Preferred Reporting Items for Systematic Reviews and Meta-Analyses (PRISMA). The review protocol was registered in the International Prospective Register of Systematic Reviews (PROSPERO) (Registration number CRD420251183772). The PRISMA Checklist is provided in the Supplementary Materials.

After the review had been registered in PROSPERO, the search was expanded from the originally planned databases (PubMed, Google Scholar, ClinicalKey) to include Web of Science and Cochrane Library. This ensured a comprehensive and reproducible methodological quality. No other changes from the registered protocol occurred.

### 2.2. Eligibility Criteria

The study was constructed according to the PRISMA guidelines; hence, the Population, Intervention, Comparison, Outcome, and Study design (PICOS) framework was applied. Randomised, retrospective, and prospective studies, case reports, and series (S) on human patients diagnosed with CLP regardless of age, gender, and ethnicity (P) were included. The included intervention for the population was TAT performed in or adjacent to the cleft region, with or without prior alveolar cleft reconstruction or bone grafting procedures (I). The outcomes (O) considered for the analysis were survival rates and radiological examination findings (e.g., root resorption/development, pulp obliteration, and ankylosis), tooth mobility test, percussion test, gingival examinations (e.g., health, plaque, depth, bleeding), and aesthetic evaluation indicating the success of the provided treatment. In addition, clinical treatment methods and their choice were observed, namely, alveolar cleft closure method, donor tooth type and root maturity, TAT recipient site, orthodontic treatment application and initiation time point, endodontic treatment application, age at alveolar cleft closure and TAT, time from bone graft to TAT, and follow-up duration. No comparison (C) was available because all included studies were single-arm case reports or case series. Therefore, outcomes were synthesised descriptively across studies. The developed focus question was: What are the treatment strategies, methodological characteristics, and clinical outcomes of TAT in patients with CLP, and what preliminary clinical treatment algorithm can be proposed based on the available evidence?

### 2.3. Search Strategy, Information Sources, and Study Selection

An advanced literature search was conducted across PubMed, Google Scholar, ClinicalKey, Web of Science, and Cochrane Library databases with no lower date limit, up to 21 June 2026. The databases were scanned using keyword-Boolean combinations as provided in Supplementary Table S1. Supplementary database Google Scholar results were sorted by relevance, and 100 accessible pages (1000 articles) were screened by title and abstract. Google Scholar is acknowledged as less reproducible due to the lack of support for field tags and limited retrievability; accordingly, a simplified PubMed Boolean string was used, and the database’s default settings were applied with no personalised account signed in. ClinicalKey was searched through the Lithuanian University of Health Sciences institutional access with the “Full Text Only” filter applied and sorted by relevance. All retrieved records were screened by title and abstract on a single day (21 June 2026), and ineligible records were excluded.

Before the initial database search, seven authors reviewed the search strategy. Further, searching, pooling, and scanning of the studies were done to exclude duplicates. Then, the study titles and abstracts were screened for relevance to the review’s criteria. For the remaining studies, full texts were carefully reviewed to confirm conformity with the requirements, and, in case of exclusion, the reason was noted. Lastly, the reference lists of the eligible studies were manually scanned to detect possible additional articles (Supplementary Table S1). The whole procedure was completed independently by two authors (M.A.S. and B.H.). Inquiries during the process were resolved by two authors (G.J. and A.V.).

### 2.4. Inclusion and Exclusion Criteria

Inclusion and exclusion criteria are presented in Table 1.

The present review addresses TAT in patients with CLP broadly, across cleft types (including cleft palate) and regardless of grafting status, with recipient sites in or adjacent to the cleft area, rather than being restricted to grafted alveolar cleft sites. Articles not reporting orthodontics and/or endodontics treatment or follow-up were excluded. This is because criteria were pre-determined in PROSPERO as main elements of both the management pathway and the primary outcome. Survival can’t be valuably explained without a defined follow-up duration, and the review’s aim highlights treatment strategies, which can’t be described if their application is unstated. These elements were set as eligibility criteria rather than data extraction variables because a study missing them can’t support the review’s outcome interpretation or treatment strategy aim, rather than leaving a data gap.

### 2.5. Data Extraction

Data extraction was performed independently by two authors (M.A.S., B.H.) using a standardised data extraction form developed for this review. The following outcome measures were extracted from the included studies.

Survival was defined as retention of the auto-transplanted tooth in situ at the last reported follow-up, regardless of the presence or absence of other clinical findings. Success was considered a broader outcome requiring favourable clinical and radiographic findings, including tooth retention, absence of major complications (e.g., root resorption, ankylosis, pathological mobility or extraction), and satisfactory periodontal, functional and aesthetic outcomes when reported. Any disagreements were resolved through discussion with two additional authors (G.J., A.V.).

When outcome data were not provided in the publication, they were recorded as “not reported” (NR). No assumptions were made regarding the presence or absence of complications when information was unavailable.

### 2.6. Risk-of-Bias/Critical Appraisal Assessment

The risk-of-bias of the included case series and reports [15,16,17,18,19,20,21,22] was evaluated in accordance with the Joanna Briggs Institute (JBI) Critical Appraisal Checklist for Case Reports and Case Series [23]. The JBI risk-of-bias scale for case series and reports consists of ten and eight domains, respectively. Each domain is deemed as “yes” (green), “no” (red), “unclear” (yellow), or “not applicable” (grey). The greater the number of domains graded as “yes”, the lower the risk-of-bias.

## 3. Results

### 3.1. Study Selection

**Figure 1:**
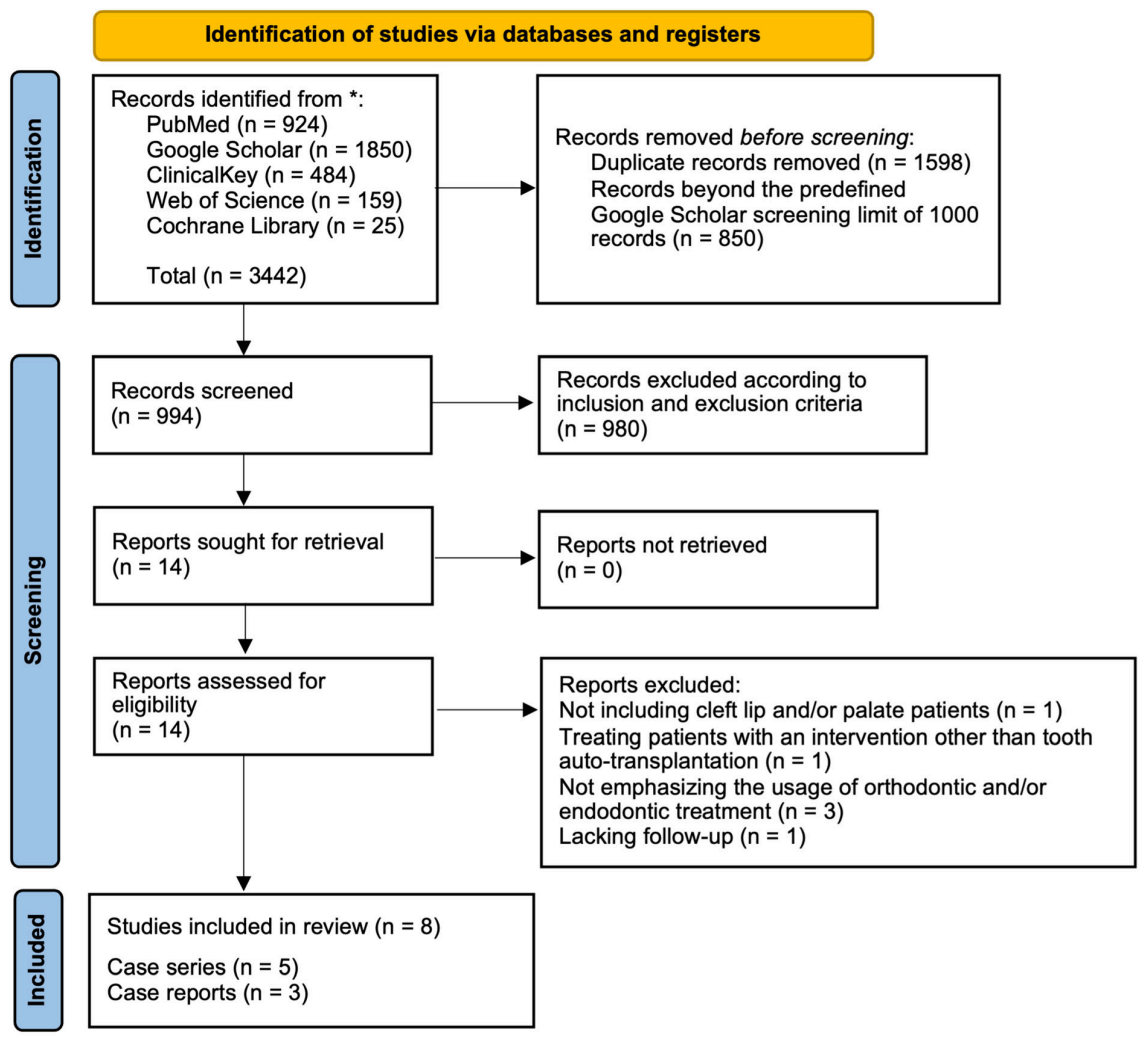
PRISMA flow diagram. * Consider, if feasible to do so, reporting the number of records identified from each database or register searched (rather than the total number across all databases/registers).

The literature review was conducted using five databases: PubMed, Google Scholar, ClinicalKey, Web of Science, and Cochrane Library. Specific keyword strings were used, and a total of 3442 articles were identified, of which 1598 were eliminated as duplicates and 850 based on Google Scholar’s 1000 articles limitation, leaving 994 studies for screening. After preliminary screening, 980 studies were excluded according to the inclusion and exclusion criteria, resulting in 14 articles eligible for full-text review. Following full-text assessment, 6 articles were excluded (detailed reasons in Supplementary Table S2), leaving 8 studies that met the inclusion criteria. Publications were released between 1998 and 2024. The search process and study selection are illustrated in the PRISMA flow diagram (Figure 1).

### 3.2. Risk-of-Bias/Critical Appraisal of the Included Studies

**Figure 2:**
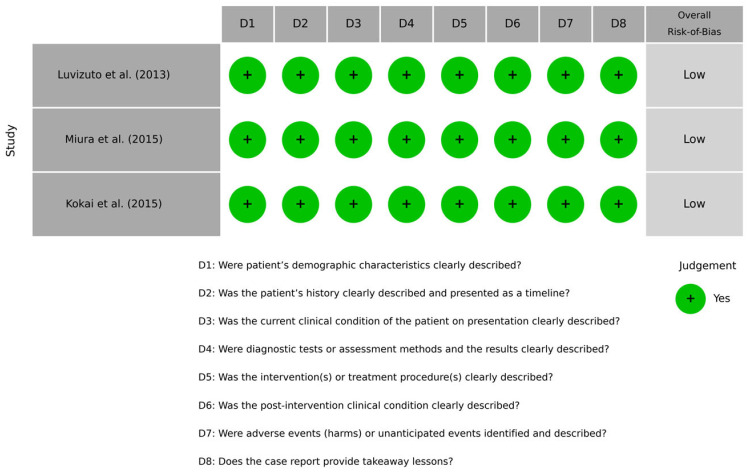
JBI critical appraisal tool evaluation for case reports [19,20,21].

**Figure 3:**
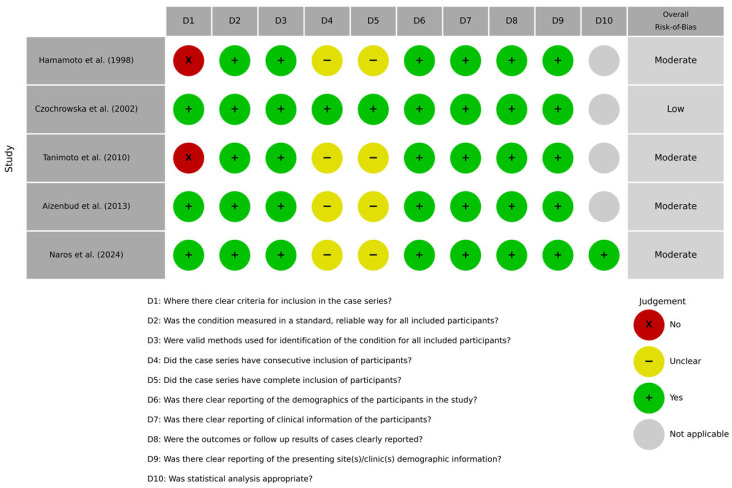
JBI critical appraisal tool evaluation for case series [15,16,17,18,22].

Based on JBI critical appraisal tools for case reports and case series, the domain-level assessments are presented in Figure 2 and Figure 3, respectively.

Overall, all studies adequately described patient characteristics, clinical history, intervention procedures, and treatment outcomes [19,20,21,22]. However, several studies demonstrated limitations related to incomplete or unclear inclusion of case reports or participants into case series [15,17,18,22]. All three case reports [19,20,21] exhibited a low risk-of-bias. Regarding case series, four [15,17,18,22] were deemed as moderate risk-of-bias, and one [16] as low risk-of-bias. Nevertheless, it should be acknowledged that all included studies were case reports or case series, which are inherently susceptible to selection bias, reporting bias and limited generalizability. The JBI checklists appraise reporting completeness rather than internal validity in the traditional sense; consequently, “low” and “moderate” ratings denote the sufficiency of reporting, not low risk of bias, and all included articles remain intrinsically at high risk of bias by virtue of their uncontrolled design. Despite the fact that the studies fulfilled most of the JBI appraisal criteria and the inability to apply Grading of Recommendations, Assessment, Development, and Evaluation Analysis (GRADE) to case reports and series, the certainty of evidence should be interpreted as “very low” due to study design, small sample size, indirectness, heterogeneity, selective reporting, and lack of comparative data for clinical decision-making.

### 3.3. Characteristics of Included Studies

The systematic review included eight studies, consisting of five case series [15,16,17,18,22] and three case reports [19,20,21]. In total, the studies comprised 23 CLP patients with a total of 27 auto-transplanted teeth. Gender-wise, 15 of the patients were males, and the remaining 8 were females. The age at alveolar cleft closure by a bone graft ranged from 9 years and 1 month to 23 years and 8 months. The time from bone graft to TAT ranged from 0 to 52 months. Patient age at TAT varied from 10 years and 5 months to 27 years and 3 months.

Methodologically, 16 out of 23 patients underwent an iliac crest bone graft for the alveolar cleft closure, specifically: secondary alveolar bone graft (SABG) with iliac crest particulate cancellous bone and marrow (PCBM) (*n* = 6); SABG with iliac crest cancellous bone (*n* = 5); SABG with autogenous iliac bone (*n* = 3); SABG with iliac crest PCBM + platelet-rich plasma (PRP) (*n* = 1); and SABG with iliac crest cancellous bone + premaxillary osteotomy (*n* = 1), and the rest were resolved conservatively without a graft [15,16,17,18,19,20,21,22]. Among all cases [15,16,17,18,19,20,21,22], mandibular 1st (*n* = 7) and 2nd (*n* = 13) premolars were the most commonly auto-transplanted, followed by maxillary 1st (*n* = 2) and 2nd (*n* = 2) premolars, maxillary 3rd molar (*n* = 1), mandibular central incisor (*n* = 1), and supernumerary tooth (*n* = 1). As for the recipient sites, the maxillary 2nd premolar was the most common (*n* = 9), followed by maxillary central incisors (*n* = 8), lateral incisors (*n* = 7), 1st premolar (*n* = 2), and canine (*n* = 1). Five studies [16,17,18,19,22] auto-transplanted teeth with immature roots (*n* = 23), while three studies [15,20,21] used teeth with mature roots (*n* = 4).

Additionally, all studies [15,16,17,18,19,20,21,22] applied orthodontic movements to the auto-transplanted teeth. However, this was not universal for all individual cases, as one patient (Case #21) in the study by Naros et al. did not undergo orthodontic treatment [22]. The application of orthodontic forces post TAT ranged from 15 days to 1 year or was initiated after healing. Endodontically, treatment implementation ranged from immediately before TAT up until 1 year post-operatively in five studies [15,17,19,21,22].

Follow-up duration was reported for all 27 auto-transplanted teeth and ranged from 10 months to 12 years and 9 months (median 5 years and 8 months). Three teeth (11.1%) were followed for less than 2 years, eight teeth (29.6%) followed for 2 to 4.9 years, twelve teeth (44.4%) for 5 to 9.9 years, and four teeth (14.8%) for 10 years or more. Across all of them, 16 teeth (59.3%) had at least 5 years of follow-up (Supplementary Table S3).

To accurately evaluate the strength of current clinical evidence, the completeness of the reported data across the eight included studies was assessed. Eight cases from the Naros et al. study failed to report the exact timing of the RCT. Nine cases from the same series vaguely stated that orthodontic treatment began after healing without specifying the exact days, weeks or months [22].

#### 3.3.1. Survival of the Auto-Transplanted Teeth

Among the included studies [15,16,17,18,19,20,21,22], with a total of 27 auto-transplanted teeth, 26 teeth remained in situ during the reported follow-up period (descriptive survival proportion: 96.3%). Only one auto-transplanted tooth (3.7%) failed and had to be extracted after 27 months due to external root resorption [22]. For the subset of teeth with sufficient follow-up, survival was 100% at 1 year (26/26 teeth) and 94.1% at 5 years (16/17 teeth). These proportions are descriptive and should not be read as estimates of treatment effectiveness, given the uncontrolled study designs and variable follow-ups. Functional, aesthetic, and occlusal outcomes were variably reported across studies and therefore could not be consistently synthesised. Success rates could not be calculated because full criteria were not evaluated in all studies. Therefore, survival is the primary outcome, though it reflects only retention and may overestimate favourable outcomes.

#### 3.3.2. Radiological Examination Findings

Upon radiological follow-up evaluation, all studies [15,16,17,18,19,20,21,22] reported a variety of positive diagnostic outcomes: periodontal space regeneration; presence of continuous lamina dura; a crown-root ratio >1; continued root length development; and bone graft radiopacification. However, Czochrowska et al., Aizenbud et al., and eight out of ten auto-transplanted teeth in Naros et al. demonstrated pulp obliteration [16,18,22].

More specifically, in the Naros et al. study, one auto-transplanted tooth did not undergo any root development and showed pathological periodontal space progression with external root resorption, while another auto-transplanted tooth demonstrated moderate root development [22].

#### 3.3.3. Tooth Mobility/Stability

A total of six articles reported the mobility and stability of 22 auto-transplanted teeth [16,17,18,20,21,22]. Twenty-one teeth (95.5%) demonstrated physiological mobility and stability, while one (4.5%) had increased mobility at the follow-up appointment [16].

#### 3.3.4. Percussion Test

Naros et al. performed a percussion test on the auto-transplanted teeth, in which one tooth responded positively with a dull sound [22].

#### 3.3.5. Gingival Examination

Gingival observations of 22 auto-transplanted teeth were reported post-follow-up in a total of five studies [16,18,20,21,22]. Aizenbud et al. and Miura et al. reported normal gingival contour, with no infections, disease, or redness around all auto-transplanted teeth [18,20]. In Naros et al., probing depth of auto-transplanted teeth did not exceed 3 mm [22].

Czochrowska et al. noted plaque accumulation on three out of five auto-transplanted teeth, yet no difference was observed in the gingival index score compared with the control group teeth. No periodontal pockets deeper than 4 mm were observed. However, in one case, the interproximal gingival papilla adjacent to the auto-transplanted tooth did not reach the contact point of the adjacent tooth. Nevertheless, the remaining auto-transplanted teeth had well-preserved papillae [16].

Additionally, Kokai et al. detected gingival recession of the buccal gingiva [21].

#### 3.3.6. Aesthetics Appraisal

Czochrowska et al. performed a structured comparative aesthetic evaluation of their five TAT cases by a professional and the involved patient in relation to the contralateral central incisor [16]. The professional rated three cases as a match and the other two as a deviation. Meanwhile, four patients were satisfied, and one was dissatisfied.

Other studies [18,19,21] qualitatively mentioned that appropriate facial and smile aesthetics of the auto-transplanted teeth had been achieved and agreed upon by the corresponding authors and/or patients.

Overall, across the parameters that were assessed: 21/22 teeth (95.5%) demonstrated physiological mobility, the gingival health of 22 teeth was adequate (isolated findings of one deficient papilla, one buccal recession, and plaque without attachment loss in three), and aesthetics were appropriate in most cases (Czochrowska et al.: professional: 3/5 match, patients: 4/5 satisfied; Aizenbud et al., Luvizuto et al., Miura et al. qualitatively reported appropriate facial and smile aesthetics). A single percussion-tested failure tooth had a positive dull sound, corresponding to peri-apical pathology. Denominators differ because each parameter was reported by a different subset of studies.

A concise overview of the characteristics and outcomes of the included studies is presented in Table 2.

Additionally, a detailed overview of characteristics and clinical outcomes is available in Supplementary Tables S3 and S4, respectively.

## 4. Discussion

The present systematic review aimed to analyse the existing literature on TAT in patients with CLP, focusing on patient characteristics, treatment strategies, and clinical outcomes, and to suggest a preliminary expert-informed clinical algorithm for their management. The results suggest that TAT has been reported with favourable outcomes in CLP patients, though the evidence is descriptive and uncontrolled.

### 4.1. Preliminary Expert-Informed Clinical Algorithm for the Treatment of Cleft Lip and/or Palate Patients with Tooth Auto-Transplantation

**Figure 4:**
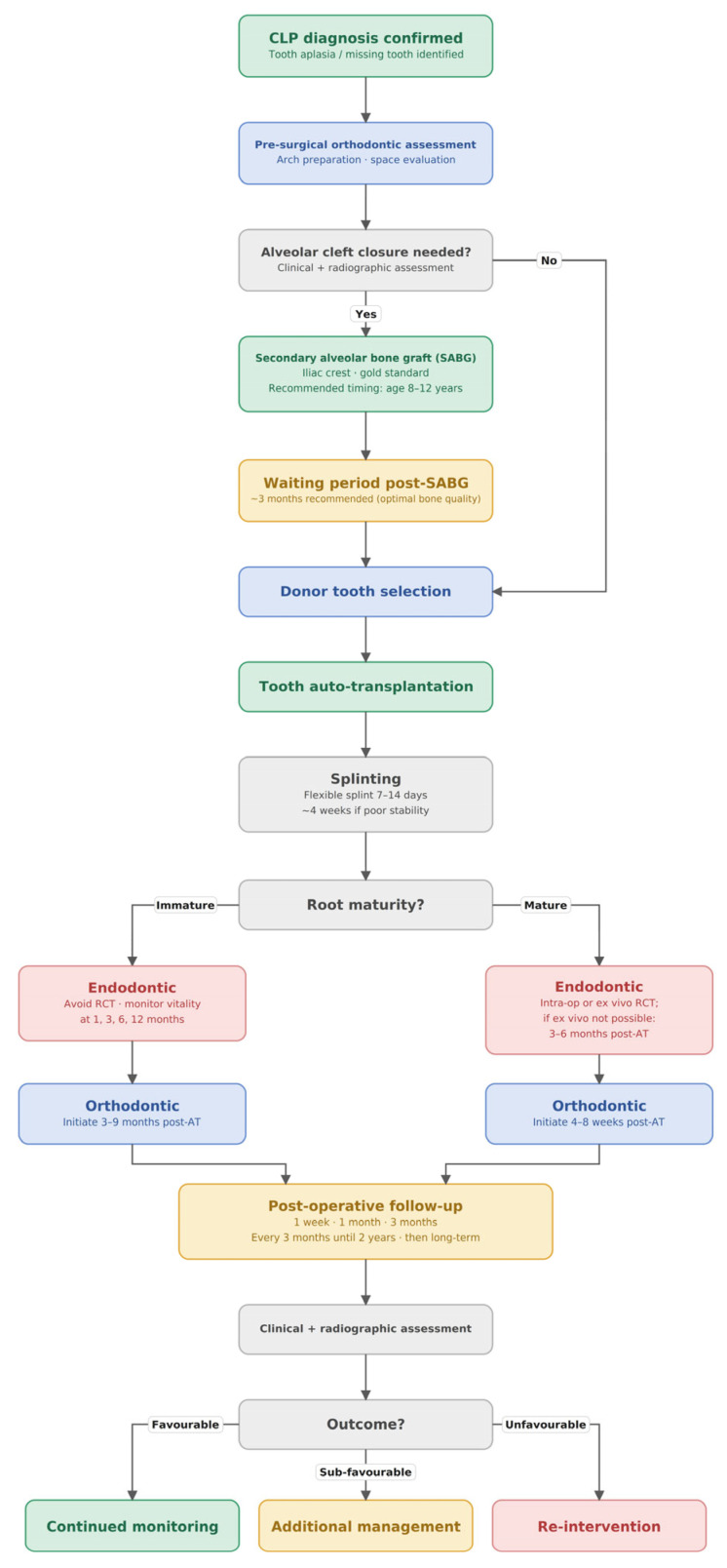
A preliminary expert-informed clinical algorithm for the management of cleft lip and/or palate patients with tooth auto-transplantation. The present algorithm is hypothesis-generating and has not undergone prospective validation. The 8–12-year SABG window is the traditional gold standard; successful outcomes have been reported at older ages. Timing parameters should be individualised based on canine eruption, tooth development, and other patient-specific factors rather than applied as rigid intervals. CLP = Cleft lip and/or palate; SABG = Secondary alveolar bone graft; RCT = Root canal treatment.

Based on the findings of the present systematic review and supported by evidence from related literature, we propose a preliminary, expert-informed clinical algorithm for the management of patients with CLP undergoing TAT, which requires prospective validation. It is illustrated in Figure 4. Separate comprehensive sections about each stage of the treatment algorithm are available below in the discussion section.

To ensure clinical safety and academic transparency, it is critical to distinguish between findings directly observed in our cleft cohort, recommendations extrapolated from broader adjacent literature, and expert clinical interpretations by the authors. Therefore, Table 3 outlines the explicit source derivation and evidence level for each core element of the proposed preliminary algorithm. Particularly, except for donor tooth type and recipient site, all timing parameters (SABG, post-graft healing period, splinting duration, endodontic and orthodontic initiation, and follow-up) are extrapolated from adjacent literature rather than from the included CLP studies and are therefore hypothesis-generating.

### 4.2. Secondary Alveolar Bone Graft

Given this complexity, the success of TAT in patients with CLP is multi-factorial, where interdisciplinary decision-making and patient-specific factors contribute to the outcome. Among these factors, the choice of alveolar cleft closure is one of the earliest determinants of success, as all the included studies [15,16,17,18,19,20,21,22] performed the gold standard SABG using the iliac crest as the donor site for patients requiring alveolar cleft closure pre-operatively. Notably, Miura et al. used PRP as an adjuvant to the iliac bone graft, aiming to promote new bone formation [20]. However, a systematic review by Vishva et al. examining the effect of PRP on bone volume following SABG in alveolar cleft patients found no statistically significant difference between PRP and non-PRP groups [35]. In addition, a similar systematic review by Thangarajah et al. concluded that PRP, platelet-rich fibrin, and control groups had comparable bone height, density, and volume [36]. Furthermore, Kokai et al. performed a premaxillary osteotomy in combination with an iliac bone graft in an adult patient aged 26 years and 6 months, with a bilateral CLP [21]. According to the literature, the surgical management of the premaxilla is advised between the ages of 8 and 12 due to numerous advantages (e.g., bone height, stability) [37]. Despite the limited evidence supporting this procedure in adult patients, Kokai et al. noted a favourable outcome [21].

Seven of the 23 patients underwent TAT without a SABG (Czochrowska et al., *n* = 2; Naros et al., *n* = 5) [16,22], where native bone was sufficient; all non-grafted teeth survived. In particular, the single failure occurred in a grafted tooth, not a non-grafted one. No study reported a bone volume threshold to omit SABG; therefore, no bypass indication could be defined, and grafting remained as the algorithm’s default, which is a gap for future study.

### 4.3. Timing of Alveolar Cleft Closure

The timing of alveolar cleft closure remains debatable to this day, as agreed by many review studies [24,25,38,39,40]. For instance, Kaura et al. determined that no chronological age had a superior outcome; however, SABG during the mixed dentition period is the consensus [38]. Further, Elhaddaoui et al. and Kim et al. stated that SABG between the ages of 8 and 12, before or just after canine eruption, is the traditional gold standard for an optimal outcome, based on the original inventors [24,25,26]. More recently, Kim et al., Fahradyan et al., and Mundra et al. noted that early mixed dentition SABG around 6 and up until 8 years of age is gaining interest; however, it may only be applied in the presence of a lateral incisor germ or just before the eruption of the central incisor. Conversely, in the case of the absence of a lateral incisor, there will be no benefits, as the grafted bone may only be preserved through the tooth eruption physiology, and alternatively the timing should be just before the eruption of the canine [25,39,40]. If alveolar cleft management is indicated during early mixed dentition, based on their clinical experience, Mundra et al. suggest a pre-operative and a 6-month post-operative orthodontic treatment [40]. Lastly, the authors acknowledge that existing data are not sufficient for definitive conclusions, and success is more related to peri-operative protocols, surgical technique, and multidisciplinary co-operation [24,25,38,39,40]. Comparatively, according to Table 2, TAT #1, #2, #16, #19, and #20, the alveolar clefts of these patients were closed at >12 years of age [15,21,22]. Despite that, these teeth survived, which may be explained by the fact that chronological age is not a definitive determinant of success, but is based on multiple factors as mentioned previously.

### 4.4. Timing of Tooth Auto-Transplantation Post Secondary Alveolar Bone Graft

Further, the waiting time from SABG to the TAT varied across the included studies [15,16,17,18,19,20,21,22]. While the current literature lacks evidence regarding the optimal timing for TAT post SABG, there is only adjacent evidence regarding the usage of cone-beam computed tomography (CBCT) for the assessment of bone quality post-grafting of alveolar clefts [27,28,41,42]. Zhang et al. and Kumar et al. observed equivalent bone height and mineral bone density to the normal anterior maxillary bone at 3 months follow-up [27,28]. Later, from 3 to 6 months, Zhang et al. noticed sustained bone density, but a decline in bone height both labially and palatally [28]. Further, studies with longer follow-up noted a periodic gradual increase in bone resorption at 6 months, 1 to 2 years, and more than 2 years [41,42]. Based on the SABG findings, a 3-month post-grafting interval may represent a biologically plausible time point for performing TAT. Among the included studies, TAT #19 and #20 had a 3-month waiting time, while the others had either less or more than this interval [15,16,17,18,19,20,21,22]. Regardless, all teeth survived, except for TAT #21, possibly indicating the multifactorial concept of success.

### 4.5. Age at Tooth Auto-Transplantation

TAT has been performed at a wide age range [15,16,17,18,19,20,21,22]. While there is no strict age limit, the operation is usually performed in patients under the age of eighteen. According to the modern literature, success rates may be associated with the age of the patient [43,44,45]. In a study by Tsukiboshi et al., a higher success rate (~92%) was observed in patients younger than 30 years, compared with patients older than 30 years (~80%) [43]. A systematic review of children/adolescents, including patients aged 8–18 years, observed an ~85% success rate and ~94% survival rate [44]. In general, the younger the patient, the better the prognosis, as supported by data from individual studies [43,44,45]. This may be explained by the superior periodontal healing potential of younger developing patients, and high pulp vascularisation in immature teeth [45].

Regarding our cases, most patients underwent TAT in adolescence, and all teeth survived at follow-up [15,16,17,18,19,20,22]. In contrast, in patients #17 and #20, TAT was done at 27 and 23 years of age, respectively, and the teeth survived despite being outside the paediatric/adolescent window, possibly attributable to other favourable factors for success (e.g., aseptic, atraumatic surgical technique) [21,22].

### 4.6. Donor Tooth Type, Root Maturity, and Recipient Site of Auto-Transplanted Teeth

Another factor which may determine the success of TAT is the donor tooth type. Accordingly, numerous systematic reviews [13,46,47] consistently deemed premolars as donors with the highest success rate and predictability, followed by canines, molars, and 3rd molars. Regardless of tooth type, root maturity is a crucial element of TAT. Based on that, teeth with immature roots (open apex) may have a greater chance of success and survival, in comparison to mature roots (closed apex), as they are associated with a higher risk of ankylosis, root resorption, and pulp necrosis [44,46,47,48,49,50,51]. Additionally, several debates exist as to whether the recipient site is a prognostic factor for TAT. Taken together, the evidence suggests that anterior recipient sites may have slightly higher success and survival rates than posterior sites [46,47,48,49,52]. This could be explained by the reduced occlusal loads, easy surgical access, and ease of hygiene maintenance in the anterior region. Overall, the authors emphasise that such findings are not consistent or well isolated and should be interpreted with caution [46,47,48,49,52]. The predominant use of premolars as donor teeth, immature roots, and the natural anterior region cleft recipient sites may explain the 96.3% survival proportion in the present study. However, this proportion pools teeth followed from 10 months to over 12 years, with only 59.3% reaching 5 years; therefore, it could overstate the long-term survival, as root resorption and pulp necrosis may occur late.

### 4.7. Endodontic Treatment—Application and Timing

As a precaution against complications in the auto-transplanted teeth, RCT is usually performed. In this regard, a systematic review by Yang et al. on the efficacy of RCT in auto-transplanted third molars noted a reduced complication rate and an improvement in long-term survival [31]. Therefore, the authors proposed a protocol based on pulpal regeneration potential [31]. In the case of mature roots, an immediate intra-operative or ex vivo RCT could be performed, and if an adequate ex vivo management environment is not possible (e.g., moisture, atraumatic handling), the RCT may be performed 3 to 6 months post TAT, during initial osseointegration [31]. Conversely, for immature roots, root canal therapy may be avoided, with careful vitality observation at 1, 3, 6, and 12 months [31]. For such teeth, endodontic management is indicated in the case of no pulp vitality recovery at six months and radiographic presence of periapical pathology [31]. Based on the proposed protocol, only the study by Kokai et al. [21] appears consistent, while other studies do not, which may be explained by the outdated nature of the protocol [15,17,19,22]. Nevertheless, all teeth survived.

### 4.8. Splinting of Auto-Transplanted Teeth

Hypothetically, the splinting method may significantly impact the success of auto-transplanted teeth. The type and/or duration of splinting were not specified in most of the included studies, with only a few studies reporting this [17,18,19,20,22]. Tanimoto et al., Luvizuto et al., and Miura et al. applied orthodontic brackets with a wire [17,19,20]. Aizenbud et al. used silk 3/0 sutures up to 2 weeks [18]. Naros et al. splinted using either titanium-trauma-splints or sutures for 10 to 26 days [22]. The evidence suggests that rigid and prolonged splinting is associated with a higher risk of ankylosis and pulp necrosis; on the contrary, flexible, short-term splinting (7–14 days) may lead to more desirable outcomes and lower failure rates [29,48]. This could be explained by the fact that rigid splinting may prevent physiological movements of the auto-transplanted tooth that allow periodontal ligament healing and pulp revitalisation [29,30]. Thus, in the case of poor stability of the auto-transplanted tooth, longer splinting (~4 weeks) may be needed [30]. Overall, the findings appear to be consistent with the included studies’ splinting methods [17,18,19,20,22].

### 4.9. Orthodontic Treatment—Application and Timing

Early application of orthodontic forces may be associated with higher success rates and lower ankylosis risk [53,54]. However, the timing may differ depending on the root formation; i.e., for teeth with mature roots, orthodontic forces may be applied 4 to 8 weeks after TAT [53]. By contrast, for teeth with immature roots, the timing in adjacent literature is 3 to 9 months post-TAT, allowing periodontal healing and pulp revascularisation, just before pulp obliteration [32,33]. Additionally, delayed orthodontic treatment may predispose the auto-transplanted teeth to external root resorption [55]. In the present review, there was great variability in the timing of orthodontic treatment for the auto-transplanted teeth (15 days to 1 year or after healing) [15,16,17,18,19,20,21,22]. Only Czochrowska et al., Miura et al., and Kokai et al. had a timing consistent with adjacent literature on orthodontic forces based on root development [16,20,21]. Yet, the other included studies did not have a strict protocol according to the literature, and despite that, favourable outcomes were reported [15,17,18,19,22].

### 4.10. Follow-Up—Duration and Examination

In the present review, among the included articles, there was great variability in follow-up durations and observations [15,16,17,18,19,20,21,22]. A retrospective cohort study of 134 auto-transplanted teeth by Marton et al. [34] noted that half of all complications may occur during the first 18 months, with the initial diagnosis at ~12–22 months:

- Inflammatory root resorption at ~12 months;
- Cervical resorption at ~18 months;
- Apical pathology at ~20 months;
- Replacement resorption at ~21 months.

Therefore, the authors [34] suggest the follow-up of auto-transplanted teeth, post-operatively at:

- 1 week;
- 1 month;
- 3 months;
- Every 3 months until 2 years;
- Continuous long-term monitoring in case of relapse or delayed complications.

Clinical and radiological assessments may be performed at each follow-up visit [18,19,22,34]. In our study, only Luvizuto et al. had adequate periodic follow-up examinations, while the others reported the follow-up at a long-term time point [15,16,17,18,19,20,21,22]. Hence, none of the studies documented a thorough evaluation of the cases [15,16,17,18,19,20,21,22]. Combining all the parameters that were observed in the included studies [15,16,17,18,19,20,21,22], we prepared a list of plausible assessments as illustrated in Table 4.

### 4.11. Radiographic and Clinical Follow-Up Determinants

TAT literature data on pulp canal obliteration indicate it is a common radiographic finding of successful pulp recovery, which may be observed partially at 6 months [56,57,58,59]. Favourable periodontal ligament healing is crucial, as it may be completed approximately after 8 weeks [59]. Radiographically, the regeneration is characterised by the presence of normal periodontal ligament space, a continuous lamina dura, and the absence of root resorption [60]. Overall, the radiographic findings of the present review are consistent with the existing literature, except for Naros et al. in TAT case #21, which exhibited external root resorption with no root development [22]. The prevalence of these complications is ~4.4% and ~4%, respectively [51,60].

Further, most of the successfully auto-transplanted teeth exhibit normal physiological mobility and stability levels at the long-term follow-up, comparable to natural teeth [34,56]. Thus, it is highly dependent on the periodontal ligament regeneration [56]. In the present study, only TAT #5 manifested increased mobility, which was deemed to be due to ~3 mm root resorption during orthodontic treatment [16].

Another key success criterion is the percussion test. A favourable outcome would be the absence of pain to percussion and the production of a low-pitched dull sound, due to the periodontal ligament cushioning effect [61]. However, in case of ankylosis, a high-pitched metallic sound would be noted, which occurs in 4.4–6.7% of auto-transplanted teeth [51,61]. Consequently, only TAT #21’s percussion test was faintly positive, indicating pain due to peri-apical root pathology and not ankylosis, as the authors stated [22].

In successfully auto-transplanted teeth, the expected probing depth varies from 2.7 to 3.0 mm, which is similar to natural teeth [62,63]. Thus, minimal gingival recession and clinical attachment level changes may be expected, yet are insignificant to consider clinically [63]. Notably, TAT #3–7 had a probing depth of no more than 4 mm, slightly exceeding the 3 mm threshold [16]. Additionally, in TAT #5, the papilla did not reach the interproximal contact point, which could be explained by the slightly increased probing depth threshold and therefore possibly indicates a distance increase from the contact point to the bony crest of more than 5 mm, leading to a significant decrease in complete papilla fill [16,64,65]. Moreover, TAT #17 had a recession in the buccal gingiva at the 3-year follow-up. Although the authors did not mention the cause, it could be due to various reasons, such as thin buccal bone, thin gingival phenotype, and more [66,67].

Nevertheless, the most important outcome for patients is aesthetics. The current evidence highlights that most cases attain a satisfactory–excellent appraisal, with 65% to 75% of auto-transplanted teeth matching or almost matching the adjacent teeth, and 71% to 89% of patients reporting a satisfactory outcome [62,68,69]. Our findings are consistent with the data from previous studies [16,18,19,21].

In total, only one TAT case failed (patient #21), and no clear reason for failure was observed when analysing the results of clinical follow-up [22]. Therefore, we suspect a possible methodological inconsistency, where unusual observations may include the absence of endodontic and orthodontic treatment of the auto-transplanted tooth. Since it has an immature root, spontaneous revascularisation may occur, but pulp necrosis may still develop, which can easily become complicated if an appropriate monitoring protocol is not followed [31,34]. Thus, the absence of orthodontic intervention may also be a partial reason for failure; as mentioned before, early timing of orthodontic forces in accordance with root formation may increase success rates significantly [53,54]. This inference is from a single failed case; thus, the algorithm’s endodontic and orthodontic timing depends on adjacent non-cleft TAT literature and this single case inference, rather than robust cleft-specific evidence. It also indicates the need for careful vitality monitoring and timely treatment, and future validation.

Barber et al. presented important prognostic factors and outcomes for TAT [70]. After synthesising the data from available studies [56,57,58,59,60,61,62,63,64,65,66,67,68,69,70] and the results of this review, we developed a list of favourable, sub-favourable, and unfavourable outcomes, which are presented in Table 5.

### 4.12. Findings of Similar Excluded Studies

Four studies reporting TAT in patients with CLP were detected during the literature search but did not qualify for our inclusion criteria [71,72,73,74]. The first original study published in 1987 by Hillerup et al. reported TAT in four CLP patients [71]. The methodology of the authors is comparable to the preliminary expert-informed clinical algorithm illustrated above. The time from SABG to TAT was more than 3 months, and a pulpectomy with calcium hydroxide deposit was performed to induce apical closure two months post-TAT. During ≥1.5-year follow-up, three of the cases were successful, and one case exhibited external inflammatory root resorption [71]. Further, the study by De Muynck et al. assessed mandibular premolar TAT in a CLP patient and reported a successful outcome after one year [72]. Consistently, Pichelmayer et al. observed favourable TAT of the mandibular 2nd premolars in bilateral CLP, following segmental distraction osteogenesis, and deemed it a viable option [73]. Lastly, Edetanlen et al. noted a successful TAT of a maxillary central incisor in a 26-year-old patient with a cleft, evidencing that optimal results may be achieved in adult patients with mature roots [74].

Overall, these excluded studies widely support the proposed algorithm: SABG-to-TAT intervals, endodontic management, and favourable premolar outcome consistent with its parameters, and Hillerup et al.’s single failure in a mature (closed-apex) tooth highlights the choice of immature donor teeth [71]. Edetanlen et al. and Kokai et al. temper the age criterion, deeming that mature root TAT in adults may still succeed and that a preference for <18 years may not be absolute [21,74].

### 4.13. Histological Findings of the Secondary Alveolar Bone Graft in Cleft Lip and/or Palate Patients

In a study by Hamamoto et al., a histological examination of the autogenous iliac bone graft was performed post TAT. The six-month specimen had a typical alveolar bone structure composed of proper cortical bone, cancellous bone, and bone marrow with regenerated blood vessels. Osteoblasts lined the bony surface, lacunae were filled with osteocytes, and tartrate-resistant acid phosphatase (TRAP)-positive osteoclasts were detected along the bone surface, while TRAP-negative multinucleated giant cells (foreign body giant cells) were not detected. The twelve-month specimen had similar characteristics, but TRAP-positive osteoclasts were seldom observed, and the ultrastructure of the bone-lining cells had poorly developed organelles.

Overall, these autogenous iliac bone findings are consistent with previous histological studies describing bone healing after TAT. One study noted that osteoclastic activity begins early and increases gradually over the course of bone healing. TRAP expression was prevalent at 28 and 60 days [75]. Osteoclastic activity transitions from active graft resorption to low-level physiological remodelling within bone multicellular units (BMUs). At 12 months, the number of active BMUs on the transplant side normalises to levels similar to those of native alveolar bone, as confirmed by CBCT showing no significant differences in bone dimensions between transplant and contralateral control sites [76,77].

### 4.14. Limitations and Recommendations for Future Research

This systematic review has several limitations that should be considered when interpreting the results. Firstly, the included studies were case reports and series, some of which lacked clearly defined and standardised treatment protocols for TAT, particularly regarding surgical, orthodontic, and endodontic management. In addition, follow-up protocols were insufficiently reported in some studies, with variability in the duration and frequency of assessments. This heterogeneity limits direct comparison of treatment outcomes, as the absence of complications during short-term follow-up cannot be interpreted as equivalent to successful long-term outcomes. Important adverse effects, including root resorption, ankylosis, pulp necrosis, and periodontal deterioration, may develop months or years after transplantation. Therefore, the reported survival and success outcomes should be interpreted in the context of follow-up duration. Furthermore, small sample sizes and the absence of randomised clinical trials reduce the level of evidence and increase the risk of bias.

The exclusion of studies not reporting orthodontics and/or endodontics treatment or follow-up may have selectively retained better reported studies; however, the excluded CLP-TAT articles summarised in Section 4.12 documented widely consistent outcomes, implying the evidence base was not materially modified.

The authors acknowledge that a formal meta-analysis, as well as subgroup and sensitivity analyses, were not suitable due to the small number of single-arm case reports and series, with heterogeneity in graft materials, donor tooth type and root maturity, the interval between SABG and TAT, orthodontic and endodontic timing, follow-up intervals, outcome interpretations, as well as incomplete reporting of some variables. Hence, the 96.3% descriptive survival proportion denotes a simple summary that does not account for the variability or missing data, rather than pooled data. Additionally, the 27 auto-transplanted teeth from 23 patients, several of whom contributed more than one tooth, means tooth-level observations are not statistically independent; this further interprets the 96.3% proportion as a descriptive summary and not as a precise estimate of treatment effectiveness. Notably, outcomes were almost uniformly favourable despite this heterogeneity, indicating that no single protocol element can be explained as determinative; the results demonstrate that TAT has been reported to succeed in various conditions rather than any specific protocol or algorithm.

These limitations highlight the need for future research with standardised protocols, larger samples, and higher-quality study designs.

## 5. Conclusions

After synthesis of available scientific data, a preliminary expert-informed clinical algorithm for patients with CLP undergoing TAT was proposed, which may become a useful tool in planning and performing this treatment procedure.

TAT may be a viable option in appropriately selected patients with CLP, demonstrating a high descriptive survival rate, which doesn’t read as an estimate of treatment efficiency, while functional and aesthetic outcomes were generally favourable in studies that assessed them. Accordingly, the following may be considered, though it is mostly extrapolated from adjacent cleft or TAT literature rather than comparative cleft and/or palate-TAT studies and therefore requiring future validation: iliac crest SABG at ≤12 years of age and 3 months of healing (if indicated); premolar donors with immature roots; anterior recipient sites; timely root maturity-based endodontic and orthodontic treatment; TAT preferably at ≤18 years of age; and consistent, thorough follow-up.

However, the available evidence is limited and heterogeneous due to small sample sizes, and the results should be interpreted with caution. Future high-quality prospective studies with long follow-ups and the application of the proposed preliminary expert-informed clinical algorithm are recommended.

## Figures and Tables

**Table 1:** Inclusion and exclusion criteria.

Inclusion Criteria	Exclusion Criteria
Articles including patients with a previous diagnosis of CLP who proceeded to TAT in or adjacent to the cleft region, with or without prior alveolar cleft reconstruction. Articles including patients with acceptable general health and no conditions that may complicate TAT. Randomized, retrospective, prospective, or case reports and series journal articles. Full-text English-language articles.	Articles observing patients with no history of CLP. Articles treating CLP patients with an innovation other than TAT. Articles involving patients with systemic health conditions that could interfere with TAT. Articles not mentioning whether orthodontic and/or endodontic treatments were applied, or the duration of follow-up post TAT. Book chapters, literature reviews, systematic reviews, and meta-analyses. Non-full-text articles published in a language other than English.

**Table 2:** A concise table presenting the characteristics and outcomes of the included studies.

Study	Study Design	Patients/Teeth	Cleft Type	Donor Tooth	Root Maturity	Grafting Approach	Graft → TAT Timing	Follow-Up	Tooth Survival	Major Complications
Hamamoto et al. (1998) [15]	Case series	2/2	UCLP	Mandibular central incisor Mandibular 2nd premolar	Mature	SABG with Iliac PCBM	6–12 m.	3–20 m.	2/2	NR
Czochrowska et al. (2002) [16]	Case series	5/5	UCLP	Mandibular 1st premolar (*n* = 4) Mandibular 2nd premolar (*n* = 1)	Immature	SABG with Iliac cancellous (*n* = 3) None (*n* = 2)	14–26 m. (*n* = 3) N/A (*n* = 2)	2.5–7.7 y.	5/5	Pulp obliteration Increased mobility (*n* = 1) Deficient papilla (*n* = 1) Patient dissatisfied (*n* = 1)
Tanimoto et al. (2010) [17]	Case series	2/2	UCLP	Maxillary 1st premolar Mandibular 1st premolar	Immature	SABG with autogenous Iliac	15–21 m.	10 m.–2.1 y.	2/2	NR
Aizenbud et al. (2013) [18]	Case series	4/5	UCLP (*n* = 2) BCLP (*n* = 2)	Mandibular 2nd premolar	Immature	SABG with Iliac PCBM	12–52 m.	2.1–6.2 y.	5/5	Pulp obliteration
Luvizuto et al. (2013) [19]	Case report	1/1	UCLP	Mandibular 2nd premolar	Immature	SABG with autogenous Iliac	6 m.	1 m.–5 y.	1/1	NR
Miura et al. (2015) [20]	Case report	1/1	BCL + UCA	Supernumerary tooth	Mature	SABG with Iliac PCBM + PRP	0 m.	1.2–2.3 y.	1/1	NR
Kokai et al. (2015) [21]	Case report	1/1	BCLP	Maxillary 2nd premolar	Mature	SABG with Iliac cancellous + Premaxillary osteotomy	9 m.	2.8–3 y.	1/1	Buccal gingival recession
Naros et al. (2024) [22]	Case series	7/10	UCLP (*n* = 6) CP (*n* = 1)	Mandibular 2nd premolar (*n* = 5) Mandibular 1st premolars (*n* = 2) Maxillary 1st premolars (*n* = 1) Maxillary 2nd premolar ( *n* = 1) Maxillary 3rd molar (*n* = 1)	Immature	SABG with Iliac cancellous (*n* = 2) None (*n* = 5)	2–3 m. (*n* = 2) N/A (*n* = 5)	2.3–12.8 y.	9/10	Pulp obliteration (*n* = 8) External root resorption, Periodontal pathology, No root development, Dull percussion (*n* = 1; tooth extraction at 27 m.) Moderate root development (*n* = 1)
Total (1998–2024) [15,16,17,18,19,20,21,22]	Case series (*n* = 5) Case report (*n* = 3)	23/27	UCLP (*n* = 18) BCLP (*n* = 3) BCL + UCA (*n* = 1) CP (*n* = 1)	Mandibular 2nd premolar (*n* = 13) Mandibular 1st premolars (*n* = 7) Maxillary 1st premolars (*n* = 2) Maxillary 2nd premolar ( *n* = 2) Maxillary 3rd molar (*n* = 1) Mandibular central incisor (*n* = 1) Supernumerary tooth (*n* = 1)	Immature (*n* = 23) Mature (*n* = 4)	SABG with Iliac PCBM (*n* = 6) SABG with Iliac cancellous (*n* = 5) SABG with autogenous Iliac (*n* = 3) SABG with Iliac PCBM + PRP (*n* = 1) SABG with Iliac cancellous + Premaxillary osteotomy (*n* = 1) None (*n* = 7)	0–52 m. N/A (*n* = 5)	1 m.–12.8 y.	26/27	Pulp obliteration (*n* = 13) Increased mobility (*n* = 1) Deficient papilla (*n* = 1) Patient dissatisfied (*n* = 1) Buccal gingival recession (*n* = 1) External root resorption, Periodontal pathology, No root development, Dull percussion (*n* = 1; tooth extraction at 27 m.) Moderate root development (*n* = 1)

SABG = Secondary alveolar bone graft; UCLP = Unilateral cleft lip and palate; BCLP = Bilateral cleft lip and palate; BCL = Bilateral cleft lip; UCA = Unilateral cleft alveolus; CP = Cleft palate; PCBM = Particulate cancellous bone and marrow; PRP = Platelet-rich plasma; NR = Not reported; y. = Year(s); m. = Month(s).

**Table 3:** Detailed source derivation and evidence level for each core element of the preliminary expert-informed clinical algorithm. All references in the “Primary Evidence Basis” column are derived from general cleft or non-cleft TAT literature and not from TAT in CLP-specific studies; the “Clinical Justification and Limitations” column reports what was demonstrated in the included CLP cohort.

Algorithm Element	Source Derivation Category	Primary Evidence Basis and References	Clinical Justification and Limitations
SABG timing (mixed dentition, 8–12 years)	Extrapolated from adjacent literature	Gold-standard cleft literature [24,25,26]	Observed in review: highly variable; several successful cases were treated at >12 years. Justification: extrapolated from established cleft timelines to optimise bone volume before canine eruption.
Post-SABG waiting period (3 months of healing)	Extrapolated from adjacent literature	Adjacent CBCT bone-density and volume literature [27,28]	Observed in review: gaps in reporting; ranged widely from 0 to 52 months. Justification: derived from adjacent radiographic data showing peak mineral bone density at 3 months post-grafting.
Splinting duration (flexible splint for 7–14 days)	Extrapolated from adjacent literature	General dental traumatology/TAT literature [29,30]	Observed in review: poorly or inconsistently specified across the included cleft studies. Justification: extrapolated to mitigate ankylosis and pulp necrosis risks associated with rigid or prolonged fixation.
Endodontic timing (immature roots: avoid RCT and observe; indicated in case of complications) (mature roots: intra-op or 3–6 months post-operatively)	Extrapolated from adjacent literature	General third-molar TAT guidelines [31]	Observed in review: highly inconsistent protocols due to the historical age of older studies. Justification: extrapolated from broader endodontic consensus to ensure systematic management of closed-apex complications.
Orthodontic timing (immature roots: indicated 3–9 months post-op) (mature roots: indicated 4–8 weeks post-op)	Extrapolated from adjacent literature	Broader biomechanical TAT studies [32,33]	Observed in review: extreme heterogeneity (ranging from 15 days to 1 year). Justification: extrapolated to allow adequate initial periodontal ligament healing and pulp revascularization before moving the tooth.
Post-op follow-up schedule (1 w., 1 m., 3 m., then every 3 m. until 2 years)	Extrapolated from adjacent literature and expert interpretation	General TAT complication timelines [34] + Author consensus	Observed in review: no included study documented a thorough, standardized, periodic tracking timeline. Justification: formulated by mapping standard clinical follow-up intervals onto the known chronological emergence of post-op complications.

SABG = Secondary alveolar bone graft; CBCT = Cone-beam computed tomography; RCT = Root canal treatment; m. = Month(s); w. = Week(s).

**Table 4:** List of clinical and radiographic examinations for auto-transplanted teeth.

Clinical Examinations	Radiographic Examinations
Tooth mobility/Stability Palpation Gingival health & periodontal probing depth Pulp vitality test Percussion test Occlusion Infection/Inflammation signs	Root development (immature teeth) Root resorption signs Periodontal ligament space Apical pathology Pulp canal obliteration Bone integration at recipient site

**Table 5:** Favourable, sub-favourable, and unfavourable follow-up outcomes in auto-transplanted teeth.

Favourable Outcomes	Sub-Favourable Outcomes *	Unfavourable Outcomes
Asymptomatic tooth Continued root development Continuous periodontal ligament and lamina dura regeneration No ankylosis Normal mobility Non-painful and low-pitch percussion Probing depth ≤ 3 mm Adequate gingival contours Papillae reaching inter-proximal contact point Matching aesthetics to adjacent/contra-lateral teeth	Pulp canal obliteration Ankylosis (high-pitch percussion) Non-filling interproximal papillae Non-matching aesthetics to adjacent/contra-lateral teeth	Symptomatic tooth Discontinued root development Discontinuous periodontal ligament and lamina dura regeneration Inflammatory root resorption Increased mobility Probing depth > 3 mm Gingival recession

* = Relatively favourable outcomes with no significant compromise on the survival of auto-transplanted teeth, which could be left as is or managed through additional interventions.

## Data Availability

No new data were created or analyzed in this study. Data sharing is not applicable in this study.

## Supplementary Materials

The following supporting information can be downloaded at: https://www.mdpi.com/article/10.3390/jcm15155829/s1, Table S1: Database search strategy; Table S2: Excluded studies reasoning; Table S3: Detailed characteristics overview; Table S4: Detailed clinical outcomes overview; File S1: PRISMA 2020 Checklist. Reference [78] is cited in the supplementary materials.

## Abbreviations

TAT	Tooth auto-transplantation
PICOS	Population, Intervention, Comparison, Outcome, and Study design
GRADE	Grading of Recommendations, Assessment, Development, and Evaluation Analysis
JBI	Joanna Briggs Institute
PRISMA	Preferred Reporting Items for Systematic Reviews and Meta-Analyses
PROSPERO	International Prospective Register of Systematic Reviews
NR	Not reported
SABG	Secondary Alveolar Bone Graft
CLP	Cleft Lip and/or Palate
PRP	Platelet-rich plasma
RCT	Root canal treatment
PCBM	Particulate cancellous bone and marrow
CBCT	Cone-beam computed tomography
TRAP	Tartrate-resistant acid phosphatase
BMU	Bone multicellular unit
