# Supplementary material for: Tooth Auto-Transplantation in Patients with Cleft Lip and/or Palate: A Systematic Review and Preliminary Clinical Protocol Proposal
Source: J Clin Med. 2026 Jul 25;15(15):5829. doi: 10.3390/jcm15155829 (PMC13467105; doi:10.3390/jcm15155829)
Supplement: Supplementary file 1 [file jcm-15-05829-s001.zip › Supplementary Table S1.pdf]

Supplementary Table S1. Overview of database search strategy, keyword-Boolean combination, and number of retrieved studies.

Date	Database	Keyword-Boolean Combination	Number of Studies
21 June 2026	PubMed	("cleft lip"[MeSH Terms] OR ("cleft"[All Fields] AND "lip"[All Fields]) OR "cleft lip"[All Fields] OR ("cleft palate"[MeSH Terms] OR ("cleft"[All Fields] AND "palate"[All Fields]) OR "cleft palate"[All Fields]) OR ("cleft"[All Fields] OR "clefted"[All Fields] OR "clefting"[All Fields] OR "clefts"[All Fields]) OR "cleft lip and palate"[All Fields] OR (("alveolar"[All Fields] OR "alveolarization"[All Fields] OR "alveolars"[All Fields]) AND ("cleft"[All Fields] OR "clefted"[All Fields] OR "clefting"[All Fields] OR "clefts"[All Fields])) OR ("orofacial"[All Fields] AND ("cleft"[All Fields] OR "clefted"[All Fields] OR "clefting"[All Fields] OR "clefts"[All Fields]))) AND ("autotransplantion"[All Fields] OR "transplantation, autologous"[MeSH Terms] OR ("transplantation"[All Fields] AND "autologous"[All Fields]) OR "autologous transplantation"[All Fields] OR "autotransplantation"[All Fields] OR "autotransplantations"[All Fields] OR ("auto"[All Fields] AND ("transplantability"[All Fields] OR "transplantable"[All Fields] OR "transplantated"[All Fields] OR "transplantating"[All Fields] OR "transplantation"[MeSH Terms] OR "transplantation"[All Fields] OR "transplantations"[All Fields] OR "transplanted"[All Fields] OR "transplanting"[All Fields] OR "transplantation"[MeSH Subheading] OR "transplantation s"[All Fields] OR "transplanter"[All Fields] OR "transplanters"[All Fields] OR "transplantion"[All Fields] OR "transplants"[MeSH Terms] OR "transplants"[All Fields] OR "transplant"[All Fields])) OR "auto-transplantation"[All Fields] OR (("teeth s"[All Fields] OR "teeths"[All Fields] OR "tooth"[MeSH Terms] OR "tooth"[All Fields] OR "teeth"[All Fields] OR "tooth s"[All Fields] OR "tooths"[All Fields]) AND ("transplantability"[All Fields] OR "transplantable"[All Fields] OR "transplantated"[All Fields] OR "transplantating"[All Fields] OR "transplantation"[MeSH Terms] OR "transplantation"[All Fields] OR "transplantations"[All Fields] OR "transplanted"[All Fields] OR "transplanting"[All Fields] OR "transplantation"[MeSH Subheading] OR "transplantation s"[All Fields] OR "transplanter"[All Fields] OR "transplanters"[All Fields] OR "transplantion"[All Fields] OR "transplants"[MeSH Terms] OR "transplants"[All Fields] OR "transplant"[All Fields])) OR (("dental health services"[MeSH Terms] OR ("dental"[All Fields] AND "health"[All Fields] AND "services"[All Fields]) OR "dental health services"[All Fields] OR "dental"[All Fields] OR "dentally"[All Fields] OR "dentals"[All Fields]) AND ("autotransplantion"[All Fields] OR "transplantation, autologous"[MeSH Terms] OR ("transplantation"[All Fields] AND "autologous"[All Fields]) OR "autologous transplantation"[All Fields] OR "autotransplantation"[All Fields] OR "autotransplantations"[All Fields])))	924

21 June 2026	Google Scholar	(cleft lip OR cleft palate OR cleft OR “cleft lip and palate” OR alveolar cleft OR orofacial cleft) AND (autotransplantation OR auto transplantation OR auto-transplantation OR tooth transplantation OR dental autotransplantation)	1850
21 June 2026	ClinicalKey	(cleft lip OR cleft palate OR cleft OR "cleft lip and palate" OR alveolar cleft OR orofacial cleft) AND (autotransplantation OR auto transplantation OR auto-transplantation OR tooth transplantation OR dental autotransplantation)<br>Filters: Source type = Full Text Only; Sort = Relevance	484
21 June 2026	Web of Science	(((((((TS = cleft lip) OR (TS = cleft palate)) OR (TS = cleft)) OR ((TS = cleft lip) AND (TS = palate))) OR (TS = alveolar cleft)) OR (TS = orofacial cleft)) AND (((((TS = autotransplantation) OR (TS = auto transplantation)) OR (TS = auto-transplantation)) OR (TS = tooth transplantation)) OR (TS = dental autotransplantation)))	159
21 June 2026	Cochrane Library	(cleft lip OR cleft palate OR cleft OR “cleft lip and palate” OR alveolar cleft OR orofacial cleft) AND (autotransplantation OR auto transplantation OR auto-transplantation OR tooth transplantation OR dental autotransplantation)	25
