# Supplementary material for: Tooth Auto-Transplantation in Patients with Cleft Lip and/or Palate: A Systematic Review and Preliminary Clinical Protocol Proposal
Source: J Clin Med. 2026 Jul 25;15(15):5829. doi: 10.3390/jcm15155829 (PMC13467105; doi:10.3390/jcm15155829)
Supplement: Supplementary file 1 [file jcm-15-05829-s001.zip › Supplementary Table S2.pdf]

Supplementary Table S2. Studies excluded in full-text screening stage with detailed reasons.

Study	Reason
Stange KM, Lindsten R, Bjerklin K. Autotransplantation of premolars to the maxillary incisor region: a long-term follow-up of 12-22 years. Eur J Orthod. 2016;38(5):508-515. doi:10.1093/ejo/cjv078	Not including cleft lip and/or palate patients
Vuletić M, Knežević P, Jokić D, Rebić J, Žabarović D, Macan D. Alveolar Bone Grafting in Cleft Patients from Bone Defect to Dental Implants. Acta Stomatol Croat. 2014 Dec;48(4):250-7. doi: 10.15644/asc47/4/2. PMID: 27688373; PMCID: PMC4872818.	Treating patients with an intervention other than tooth auto-transplantation
Hillerup S, Dahl E, Schwartz O, Hjørting-Hansen E. Tooth transplantation to bone graft in cleft alveolus. Cleft Palate J. 1987;24(2):137-141.	Not emphasizing the usage of orthodontic and/or endodontic treatment
De Muynck S, Verdonck A, Schoenaers J, Carels C. Combined surgical/orthodontic treatment and autotransplantation of a premolar in a patient with unilateral cleft lip and palate. Cleft Palate Craniofac J. 2004;41(4):447-455. doi:10.1597/03-026.1	
Edetanlen BE, Azodo CC, Egbor PE, Akpata O. Autogenous Tooth Transplantation In Adult Orofacial Cleft Deformity: A Case Report. Benin J Postgrad Med. 2009;11(1). doi:10.4314/bjpm.v11i1.48858	
Pichelmayer M, Mossböck R, Droschl H. Maxillary segmental distraction in a patient with bilateral cleft lip and alveolus with subsequent tooth transplantation: a preliminary case report. Cleft Palate Craniofac J. 2008;45(4):446-451. doi:10.1597/07-118.1	Lacking follow-up
