# Supplementary material for: Tooth Auto-Transplantation in Patients with Cleft Lip and/or Palate: A Systematic Review and Preliminary Clinical Protocol Proposal
Source: J Clin Med. 2026 Jul 25;15(15):5829. doi: 10.3390/jcm15155829 (PMC13467105; doi:10.3390/jcm15155829)
Supplement: Supplementary file 1 [file jcm-15-05829-s001.zip › Supplementary Table S3.pdf]

Supplementary Table S3. Detailed characteristics overview of the included case series & reports in a case-tooth-wise manner.

Study	Study design	Patient case number	Gender	Tooth auto-transplantation case number	Cleft type	Alveolar cleft closure method	Donor tooth type	Root maturity	Auto-transplantation recipient site	Orthodontic treatment application	Orthodontic treatment time point	Endodontic treatment application	Endodontic treatment time point	Age at alveolar cleft closure	Time from bone graft until tooth auto-transplantation	Age at tooth auto-transplantation	Follow-up duration
**Hamamoto et al. (1998) [15]**	Case series	1	Male	1	UCLP	SABG with iliac crest PCBM	Mandibular central incisor	Mature	Maxillary lateral incisor	Yes	After 7m.	Yes	After 3w.	14y.	6m.	14y. & 6m.	3m., 12m.
		2	Male	2	UCLP	SABG with iliac crest PCBM	Mandibular 2nd premolar	Mature	Maxillary lateral incisor	Yes	After 7m.	Yes	After 3w.	14y.	12m.	15y.	7m., 20m.
**Czochrowska et al. (2002) [16]**	Case series	3	Male	3	UCLP	SABG with iliac crest cancellous bone	Mandibular 1st premolar	Immature	Maxillary central incisor	Yes	After ≥ 6m.	No	N/A	9y. & 1m.	19m.	10y. & 8m.	7y. & 8m.
		4	Female	4	UCLP	SABG with iliac crest cancellous bone	Mandibular 1st premolar	Immature	Maxillary central incisor	Yes	After ≥ 6m.	No	N/A	9y. & 9m.	26m.	11y. & 11m.	6y. & 6m.
		5	Male	5	UCLP	SABG with iliac crest cancellous bone	Mandibular 1st premolar	Immature	Maxillary central incisor	Yes	After ≥ 6m.	No	N/A	9y. & 3m.	14m.	10y. & 5m.	6y. & 6m.
		6	Female	6	UCLP	None	Mandibular 1st premolar	Immature	Maxillary central incisor	Yes	After ≥ 6m.	No	N/A	N/A	N/A	11y. & 6m.	5y. & 10m.

		7	Male	7	UCLP	None	Mandibular 2nd premolar	Immature	Maxillary central incisor	Yes	After ≥ 6m.	No	N/A	N/A	N/A	13y. & 4m.	2y. & 6m.
**Tanimoto et al. (2010) [17]**	Case series	8	Male	8	UCLP	SABG with autogenous iliac bone	Maxillary 1st premolar (R)	Immature	Maxillary 2nd premolar (L)	Yes	After 15d.	Yes	After 2w.	12y. & 10m.	15m.	14y. & 1m.	2y. & 1m.
		9	Male	9	UCLP	SABG with autogenous iliac bone	Mandibular 1st premolar	Immature	Maxillary lateral incisor	Yes	After 10m.	Yes	After 2w.	12y. & 6m.	21m.	14y. & 3m.	10m.
**Aizenbud et al. (2013) [18]**	Case series	10	Male	10	UCLP	SABG with iliac crest PCBM	Mandibular 2nd premolar	Immature	Maxillary central incisor	Yes	After 6m.	No	N/A	11y. & 1m.	28m.	13y. & 5m.	6y. & 2m.
		11	Female	11	BCLP	SABG with iliac crest PCBM	Mandibular 2nd premolar	Immature	Maxillary lateral incisor	Yes	After 6m.	No	N/A	9y. & 3m.	52m.	13y. & 7m.	6y.
		12	Male	12	UCLP	SABG with iliac crest PCBM	Mandibular 2nd premolar	Immature	Maxillary canine	Yes	After 6m.	No	N/A	9y. & 1m.	47m.	13y.	2y. & 1m.

		13	Male	13	BCLP	SABG with iliac crest PCBM	Mandibular 2nd premolar (L)	Immature	Maxillary 2nd premolar (L)	Yes	After 6m.	No	N/A	12y. & 2m.	12m.	13y. & 2m.	5y. & 8m.
				14			Mandibular 2nd premolar (R)		Maxillary 2nd premolar (R)								
**Luvizuto et al. (2013) [19]**	Case report	14	Female	15	UCLP	SABG with autogenous iliac bone	Mandibular 2nd premolar	Immature	Maxillary central incisor	Yes	After 1y.	Yes	After 1y.	10y.	6m.	10y. & 6m.	1m.<br>6m.<br>1y.<br>5y.
**Miura et al. (2015) [20]**	Case report	15	Male	16	Bilateral CL + Unilateral CA	SABG with iliac crest PCBM + PRP	Supernumerary tooth (behind maxillary lateral incisor)	Mature	Maxillary lateral incisor	Yes	After 1m.	No	N/A	11y. & 10m.	0m.	11y. & 10m.	1y. & 2m.<br>2y. & 3m.
**Kokai et al. (2015) [21]**	Case report	16	Female	17	BCLP	SABG with iliac crest cancellous bone + Premaxillary osteotomy	Maxillary 2nd premolar	Mature	Maxillary lateral incisor	Yes	After 1m.	Yes	Immediately (before auto-transplantation)	26y. & 6m.	9m.	27y. & 3m.	2y. & 10m.<br>3y.
**Naros et al. (2024) [22]**	Case series	17	Female	18	UCLP	None	Mandibular 2nd premolar	Immature	Maxillary 2nd premolar	Yes	After healing	Yes	NR	N/A	N/A	12y. & 11m.	4y.
		18	Male	19	UCLP	SABG with iliac crest cancellous bone	Mandibular 2nd premolar	Immature	Maxillary central incisor	Yes	After healing	Yes	NR	15y.	3m.	15y. & 3m.	11y. & 1m.
				20			Maxillary 3rd molar		Maxillary 2nd premolar					23y. & 8m.	3m.	23y. & 11m.	2y. & 5m.

		19	Male	21	UCLP	SABG with iliac crest cancellous bone	Maxillary 2nd premolar	Immature	Maxillary lateral incisor	No	N/A	No	N/A	12y. & 1m.	2m.	12y. & 3m.	2y. & 3m.
		20	Female	22	UCLP	None	Mandibular 1st premolar	Immature	Maxillary 2nd premolar	Yes	After healing	Yes	NR	N/A	N/A	10y. & 7m.	11y. & 4m.
		21	Male	23	UCLP	None	Maxillary 1st premolar	Immature	Maxillary 1st premolar	Yes	After healing	Yes	NR	N/A	N/A	10y. & 7m.	12y. & 9m.
		22	Female	24	CP	None	Mandibular 2nd premolar	Immature	Maxillary 2nd premolar	Yes	After healing	No	NR	N/A	N/A	10y. & 7m.	10y.
				25			Mandibular 2nd premolar		Maxillary 2nd premolar			Yes				11y. & 1m.	9y. & 7m.
		23	Male	26	UCLP	None	Mandibular 2nd premolar	Immature	Maxillary 1st premolar	Yes	After healing	Yes	NR	N/A	N/A	12y. & 7m.	6y. & 8m.
				27			Mandibular 1st premolar		Maxillary 2nd premolar							13y. & 6m.	5y. & 9m.

SABG = Secondary alveolar bone graft; UCLP = Unilateral cleft lip and palate; BCLP = Bilateral cleft lip and palate; CL = Cleft lip; CA = Cleft alveolus; CP = Cleft palate; PCBM = Particulate cancellous bone and marrow; PRP = Platelet-rich plasma; N/A = Not applicable; NR = Not reported; y. = Year(s); m. = Month(s); w. = Week(s).
