# Supplementary material for: Tooth Auto-Transplantation in Patients with Cleft Lip and/or Palate: A Systematic Review and Preliminary Clinical Protocol Proposal
Source: J Clin Med. 2026 Jul 25;15(15):5829. doi: 10.3390/jcm15155829 (PMC13467105; doi:10.3390/jcm15155829)
Supplement: Supplementary file 1 [file jcm-15-05829-s001.zip › Supplementary Table S4.pdf]

Supplementary Table S4. Detailed clinical outcomes overview of the included case series & reports in a case-tooth-wise manner.

Study	Patient case number	Tooth auto-transplantation case number	Tooth survival	Radiological examination findings	Tooth mobility/stability	Percussion test	Gingival examinations	Aesthetics appraisal
**Hamamoto et al. (1998) [15]**	1	1	Yes	Positive	NR	NR	NR	NR
	2	2	Yes	Positive	NR	NR	NR	NR
**Czochrowska et al. (2002) [16]**	3	3	Yes	Positive; pulp obliteration	Normal	NR	Pocket depth ≤4 mm	Professional - Deviates from contralateral tooth<br>Patient - Satisfied
	4	4	Yes	Positive; pulp obliteration	Normal	NR	Pocket depth ≤4 mm	Professional - Matches contralateral tooth<br>Patient - Satisfied
	5	5	Yes	Positive; pulp obliteration	Increased mobility	NR	Pocket depth ≤4 mm<br>Interproximal gingival papilla not reaching contact point	Professional - Matches contralateral tooth<br>Patient - Satisfied
	6	6	Yes	Positive; pulp obliteration	Normal	NR	Pocket depth ≤4 mm	Professional - Matches contralateral tooth<br>Patient - Dissatisfied

	7	7	Yes	Positive; pulp obliteration	Normal	NR	Pocket depth ≤4 mm	Professional - Deviates from contralateral tooth<br>Patient - Satisfied
**Tanimoto et al. (2010) [17]**	8	8	Yes	Positive	Normal	NR	NR	NR
	9	9	Yes	Positive	Normal	NR	NR	NR
**Aizenbud et al. (2013) [18]**	10	10	Yes	Positive; pulp obliteration	Normal	NR	Normal gingival contours	Appropriate facial & smile aesthetics
	11	11	Yes	Positive; pulp obliteration	Normal	NR	Normal gingival contours	Appropriate facial & smile aesthetics
	12	12	Yes	Positive; pulp obliteration	Normal	NR	Normal gingival contours	Appropriate facial & smile aesthetics
	13	13	Yes	Positive; pulp obliteration	Normal	NR	Normal gingival contours	Appropriate facial & smile aesthetics

		14	Yes	Positive; pulp obliteration	Normal	NR	Normal gingival contours	Appropriate facial & smile aesthetics
**Luvizuto et al. (2013) [19]**	14	15	Yes	Positive	NR	NR	NR	Appropriate facial & smile aesthetics
**Miura et al. (2015) [20]**	15	16	Yes	Positive	Normal	NR	Normal gingival contours	NR
**Kokai et al. (2015) [21]**	16	17	Yes	Positive	Normal	NR	Buccal gingival recession	Appropriate facial & smile aesthetics
**Naros et al. (2024) [22]**	17	18	Yes	Positive; pulp obliteration	Normal	Normal	Pocket depth ≤3 mm	NR
	18	19	Yes	Positive; pulp obliteration	Normal	Normal	Pocket depth ≤3 mm	NR
		20	Yes	Positive; pulp obliteration	Normal	Normal	Pocket depth ≤3 mm	NR
	19	21	No (extracted at 27m.)	No root development Pathological periodontal progression with external root resorption	Normal	Positive / dull sound	Pocket depth ≤3 mm	NR

	20	22	Yes	Positive; pulp obliteration	Normal	Normal	Pocket depth ≤3 mm	NR
	21	23	Yes	Positive; pulp obliteration	Normal	Normal	Pocket depth ≤3 mm	NR
	22	24	Yes	Positive; moderate root development	Normal	Normal	Pocket depth ≤3 mm	NR
		25	Yes	Positive; pulp obliteration	Normal	Normal	Pocket depth ≤3 mm	NR
	23	26	Yes	Positive; pulp obliteration	Normal	Normal	Pocket depth ≤3 mm	NR
		27	Yes	Positive; pulp obliteration	Normal	Normal	Pocket depth ≤3 mm	NR

Positive = Periodontal space regeneration, presence of continuous lamina dura, a crown-root ratio >1, continued root length development, and bone graft radiopacification; m. = Month(s); NR = Not reported.
